# Perceived stress as a risk factor for peptic ulcers: a register-based cohort study

**DOI:** 10.1186/s12876-016-0554-9

**Published:** 2016-11-28

**Authors:** Ulrik Deding, Linda Ejlskov, Mads Phillip Kofoed Grabas, Berit Jamie Nielsen, Christian Torp-Pedersen, Henrik Bøggild

**Affiliations:** 1 Department of Health Science and Technology, Public Health and Epidemiology Group, Aalborg University, Niels Jernes Vej 14, Aalborg, Øst 9220 Denmark; 2 Department of Clinical Epidemiology, Aalborg University Hospital, Sdr. Skovvej 15, Aalborg, DK-9000 Denmark

**Keywords:** Peptic ulcer, Psychological stress, NSAID, Cohen’s perceived stress scale, PSS-10, Eradication therapy, Smoking

## Abstract

**Background:**

The association between stress and peptic ulcers has been questioned since the discovery of *helicobacter pylori*. This study examined whether high perceived everyday life stress was associated with an increased risk of either receiving a triple treatment or being diagnosed with a peptic ulcer.

**Methods:**

Cohen’s perceived stress scale measured the level of stress in a general health survey in 2010 of 17,525 residents of northern Jutland, Denmark, and was linked with National Danish registers on prescription drugs and hospital diagnoses. Cox proportional hazard regression was used to estimate the risk of either receiving a triple treatment or being diagnosed in a hospital with a peptic ulcer, in relation to quintiles of stress levels.

**Results:**

A total of 121 peptic ulcer incidents were recorded within 33 months of follow-up. The lowest stress group had a cumulative incidence proportion of either receiving triple treatment or being diagnosed with peptic ulcer of approximately 0.4%, whereas the highest stress group had a cumulative incidence proportion of approximately 1.2%. Compared with that of the lowest stress group, those in the highest stress group had a 2.2-fold increase in risk of either receiving triple treatment or being diagnosed with peptic ulcer (HR 2.24; CI 95% 1.16:4.35) after adjustment for age, gender, socioeconomic status, non-steroid anti-inflammatory drug use, former ulcer and health behaviours. There was no difference in risk between the four least stressed quintiles. Subgroup analysis of diagnosed peptic ulcer patients revealed the same pattern as the main analysis, although the results were not significant.

**Conclusion:**

The highest level of perceived everyday life stress raised the risk of either receiving triple treatment or being diagnosed with peptic ulcer during the following 33 months more than twice compared with that of the lowest level of perceived stress.

## Background

Since the discovery of *helicobacter pylori (H. pylori)*, the role of psychosocial factors in the development of peptic ulcers has been largely disregarded [1, 2]. Today, *H. pylori* infection [3, 4], non-steroid anti-inflammatory drug (NSAID) use [5, 6] and smoking are considered the main causes of peptic ulcers [2, 7, 8]. Thus, alternative determinants of peptic ulcers have received limited attention in recent studies. However, not all peptic ulcers can be accounted for by one of these determinants [5, 6, 9, 10]. Between 5 and 20% of peptic ulcers are idiopathic ulcers [2, 11] and the prevalence of non-*H. pylori* and non-NSAID peptic ulcers are increasing worldwide [12]. Novel research indicated that investigating an increased number of determinants could potentially provide greater insights into the mechanism behind the development of peptic ulcers [4, 7, 13]. In the literature, it was stressed that psychosocial factors, such as stress, depression and anxiety, were associated with impeded healing of duodenal ulcers [14, 15]. This suggests that these factors can influence the biological mechanisms (such as blood flow and gastric acid secretion) that can affect peptic ulcer development. This hypothesis was supported by several recent studies. In a sample of 233,093 Swedish males, decreased stress resilience significantly increased the risk of peptic ulcers [16]. Levenstein et al. [10] concluded that psychological stress increased the incidence of peptic ulcers, regardless of *H. pylori* infection or NSAID use. The authors suggested that the observed increase could partially be due to stress influencing health risk behaviours related to the development of peptic ulcers.

A number of factors have been identified as possible determinants in the development of peptic ulcers (smoking [2, 17–24], NSAID use [2, 5, 7, 17, 20], gender [9, 17, 25, 26], age [17, 21, 26], socioeconomic status [9, 25, 27–29], alcohol consumption [18, 22, 24], gastric acid secretion [3, 16], lack of sleep [18], home crowding [16], strenuous work [9, 29], family history [30] and body weight [15, 21]). Furthermore, a number of studies indicated stress or stress-related incidents as a risk factor for the development of a peptic ulcer [5, 13, 16, 17, 21, 30]. Other studies have found no evidence that peptic ulcers are a psychosomatic disorder [22, 31, 32].

No studies have included a proton pump inhibitor or H_2_-receptor antagonist, combined with two antibiotics (triple treatment) in the outcome measure. Individuals receiving this triple treatment without endoscopy or gastroscopy could be less severe cases than those tested. Therefore, this study may add some knowledge to whether the link between stress and peptic ulcer, suggested by earlier research, is also observed in this group of individuals.

The aim of this study was to examine whether a high self-perceived stress level was associated with increased risk of peptic ulcers (defined as either receiving triple treatment or being diagnosed with a peptic ulcer during follow-up).

## Method

This was a register-based cohort study linking data gathered from existing Danish registers and the North Denmark Health Profile 2010 [33]. The region of North Denmark encompassed 570,000 inhabitants. The North Denmark Health Profile 2010 was a survey whose primary aim was to describe the citizens’ health state. A questionnaire was administered to 35,700 Danish citizens over the age of 16 across 11 municipalities covering the entire northern Jutland. The data were collected from February 5^th^ to March 22^nd^, 2010. Individuals who did not respond, received two reminders by mail [33]. Cohen’s perceived stress scale (PSS-10) [34] was included in the health profile.

*The Danish Civil Registration System* included information on the unique personal identification number (CPR) that was assigned to all individuals living in Denmark [35]. The CPR numbers made it possible to link data from all included registers. CPR numbers were encrypted after linkage to maintain the respondents’ anonymity. All prescriptions redeemed in Denmark were recorded in *The Danish National Prescription Registry* with the date and ATC-codes (anatomical therapeutic chemicals) for the drugs redeemed [36]. *The National Patient Register* recorded ICD-10 codes for both somatic and psychiatric diagnoses for in- and out-patients in all hospitals, as well as the dates of hospitalization and discharge from the hospital [37]. *The Income Statistics Register,* which contained the individual incomes of the entire Danish population, was based on information from smaller registers such as The Central Taxpayers’ Register and The Salary Information Register [38]. *The Population’s Education Register* records ongoing and completed educations for all Danish citizens [39].

### Exposure

*PSS-10* [34] *score* was calculated from the answers provided in the North Denmark Health Profile 2010. PSS-10 consisted of 10 items regarding predictability, controllability and life overload, as perceived by the individual during the last month [40, 41]. Each question had five possible answers on a scale, ranging from *never* to *very often* and each item was correspondingly coded 0–4. The PSS-10 score was the total of the ten items, producing a range from 0 to 40. Respondents were subsequently divided into quintiles based on their PSS-10 score. Respondents were divided into quintiles as the PSS-10 is not a diagnostic instrument and should only be used for comparisons within a sample as there are no cut-offs [42]. The higher the PSS-10 score, the greater the respondent’s perceived feeling of psychological stress [41]. Cohen’s perceived stress scale has been validated as a measure of stress with consistent results for decades [41].

### Outcome

The treatment recommended for peptic ulcer was a triple treatment for eradication of *H. pylori*, consisting of a proton pump inhibitor (PPI) or an H_2_-receptor antagonist, combined with two antibiotics over a 7–14 day period [6, 43]. If this treatment was inefficient, an alternative combination was recommended [6, 43].

*Peptic Ulcer* was defined as either a hospital discharge diagnosis or a redeemed prescription for the triple treatment. It was coded as a dichotomous variable. Individuals who redeemed prescriptions for either a PPI or an H_2_-receptor antagonist, combined with two specific antibiotics, one macrolide and one defined as “other antibiotics” (see Table 1), were identified in the Danish National Prescription Registry. Both antibiotics had to be redeemed on the same date, whereas the PPI or H_2_-receptor antagonist could be redeemed within 60 days preceding antibiotics. Individuals who did not redeem a prescription for a macrolide, but for a PPI or H_2_-receptor antagonist combined with both amoxicillin and metronidazole or tetracycline and metronidazole were also classified as triple treated.

**Table 1:** ATC-codes for prescription drugs used to identify individuals receiving triple treatment

Drug group	Generic name	ATC-code
Proton Pump Inhibitors	Omeprazole	A02BC01
	Pantoprazole	A02BC02
	Lansoprazole	A02BC03
	Rabeprazole	A02BC04
	Ensomeprazole	A02BC05
H_2_-receptor antagonists	Cimetidine	A02BA01
	Ranitidine	A02BA02
	Famotidine	A02BA03
	Nizatidine	A02BA04
	Niperotidine	A02BA05
	Roxatidine	A02BA06
	Ranitidine Bismuth Citrate	A02BA07
	Lafutidine	A02BA08
Macrolides	Erythromycin	J01FA01
	Spiramycin	J01FA02
	Midecamycin	J01FA03
	Oleandomycin	J01FA05
	Roxithromycin	J01FA06
	Josamycin	J01FA07
	Troleandomycin	J01FA08
	Clarithromycin	J01FA09
	Azithromycin	J01FA10
	Miocamycin	J01FA11
	Rokitamycin	J01FA12
	Dirithromycin	J01FA13
	Flurithromycin	J01FA14
	Telithromycin	J01FA15
Other Antibiotics	Amoxicillin	J01CA04
	Metronidazole	J01XD01
	Metronidazole	G01AF01
	Metronidazole	P01AB01
	Metronidazole	A01AB17
	Metronidazole	D06BX01
	Tinidazole	P01AB02
	Tetracycline	D06AA04
	Tetracycline	S01AA09
	Tetracycline	S02AA08
	Tetracycline	S03AA02
	Tetracycline	J01AA07
	Tetracycline	A01AB13

To be classified as triple treatment, one drug from either the proton pump inhibitor (PPI) or H_2_-receptor antagonist group combined with one macrolide and one other antibiotic. Both antibiotics had to be redeemed on the same date, whereas the PPI or H_2_-receptor antagonist could be redeemed within 60 days preceding antibiotics. Individuals redeeming prescriptions for PPIs or H_2_-receptor antagonists combined with either amoxicillin and metronidazole or tetracycline and metronidazole were also classified as triple treated

**Table 2:** Baseline characteristics for individuals with and individuals without defined peptic ulcer during 33 months

Variable	Level	No ulcer^a^ (*n* = 17,404)	Ulcer^a^ (*n* = 121)	Total (*n* = 17525)	*P*-value
PSS-10 Group	0 - Low stress level	3497 (20.1)	13 (10.7)	3510 (20.0)	
	1	3036 (17.4)	18 (14.9)	3054 (17.4)	
	2	3517 (20.2)	17 (14.0)	3534 (20.2)	
	3	3719 (21.4)	26 (21.5)	3745 (21.4)	
	4 - High stress level	3635 (20.9)	47 (38.8)	3682 (21.0)	<0.0001
Gender	Male	8773 (50.4)	51 (42.1)	8824 (50.4)	
	Female	8631 (49.6)	70 (57.9)	8701 (49.6)	0.0855
Age	Mean (sd)	49.6 (17.1)	60.7 (15.1)	49.7 (17.1)	<0.0001
Smoking	No, never	8329 (47.9)	44 (36.4)	8373 (47.8)	
	No, but used to	5022 (28.9)	40 (33.1)	5062 (28.9)	
	Yes, <15 a day	2268 (13.0)	16 (13.2)	2284 (13.0)	
	Yes,>14 a day	1785 (10.3)	21 (17.4)	1806 (10.3)	0.0191
Alcohol consumption^b^	Over recommended	1496 (8.6)	7 (5.8)	1503 (8.6)	
	Within recommended	15908 (91.4)	114 (94.2)	16022 (91.4)	0.3486
NSAID-use	No	6792 (39.0)	27 (22.3)	6819 (38.9)	
	Yes	10612 (61.0)	94 (77.7)	10706 (61.1)	0.0002
Educational Level	Primary	5597 (32.2)	52 (43.0)	5649 (32.2)	
	Secondary	7647 (43.9)	56 (46.3)	7703 (44.0)	
	Higher	4160 (23.9)	13 (10.7)	4173 (23.8)	0.0013
Body Mass Index	Underweight	338 (1.9)	4 (3.3)	342 (2.0)	
	Normal weight	7953 (45.7)	52 (43.0)	8005 (45.7)	
	Overweight	9113 (52.4)	65 (53.7)	9178 (52.4)	0.5010
Sleep	<7 h/day	3180 (18.3)	33 (27.3)	3213 (18.3)	
	7 h/day	6729 (38.7)	29 (24.0)	6758 (38.6)	
	>7 h/day	7495 (43.1)	59 (48.8)	7554 (43.1)	0.0015
Household Income^c^	<243,646	3942 (22.6)	38 (31.4)	3980 (22.7)	
	243,646-451,597.50	4200 (24.1)	39 (32.2)	4239 (24.2)	
	451,597.50–665,147	4502 (25.9)	23 (19.0)	4525 (25.8)	
	>665,147	4760 (27.4)	21 (17.4)	4781 (27.3)	0.0030
Previous Ulcer	No previous Ulcer	16862 (96.9)	106 (87.6)	16968 (96.8)	
	Previous Ulcer	542 (3.1)	15 (12.4)	557 (3.2)	<0.0001

Baseline date was 22^nd^ of March, 2010. Follow-up was 33 months. Mean and standard deviation (sd) were reported for continuous covariates, whereas categorical covariates were described with frequencies and percentages

^a^Ulcer implies a diagnosis or triple therapy (proton inhibitor or H_2_-receptor antagonist and 2 relevant antibiotics)

^b^Recommended maximum consumption per week was 14 units for women and 21 units for men

^c^Household income was reported in Danish Kroner (DKK)

Individuals who were diagnosed in a hospital with any type of peptic ulcer were identified through the National Patient Registry. The diagnoses codes used to identify peptic ulcer patients were all ICD-10 codes [37] from K25 to K279. These codes included all types of peptic ulcers, both duodenal and gastric.

### Covariates

Because smoking [2, 17–24], NSAID use [2, 5, 7, 17, 20], gender [9, 17, 25, 26], age [17, 21, 26], socioeconomic status [9, 25, 27–29], alcohol consumption [18, 22, 24], lack of sleep [18] and body weight [15, 21] were identified in previous studies as possible determinants of peptic ulcer development, these were included in the analysis.

*Age* was included as a continuous variable and was derived from The Danish Civil Registration System [35].

*Gender* was derived from The Danish Civil Registration System [35].

*Smoking* was grouped as never smoked, former smoker, smoking 1–14 cigarettes per day and/or cheeroots, cigars or pipe bowl of tobacco daily and a group smoking more than 14 cigarettes per day. Data on smoking was computed from respondents’ answers to questions regarding their smoking in the North Denmark Health Profile 2010. The respondents were asked whether they smoked or used to smoke on a daily basis, and if they did, how many cigarettes, cheroots, cigars and pipe bowls of tobacco they smoked per day on average [33].

*NSAID* use was included as a dichotomous variable. Respondents who reported having taken non-prescription painkillers within three months preceding baseline were identified in the North Denmark Health Profile 2010 and grouped with respondents who were registered in the prescription database as having received NSAIDs within the same three months.

*Alcohol consumption* was included as a dichotomous variable based on the recommendations for moderate alcohol intake at baseline from the Danish Health Authorities [44]. Respondents were identified as having a low level of consumption (<=14 units per week for women and < =21 units per week for men) or a high level of consumption (>14 units per week for women and >21 units per week for men). One unit of alcohol corresponded to 12 g in Denmark. Alcohol consumption was calculated based on the units of alcohol per week that the respondents reported in the North Denmark Health Profile 2010.

*Body Mass Index* (BMI) was included as a categorical variable, grouped with BMI < 18.5 as underweight, BMI of 18.5–25.0 as normal weight and BMI > 25.0 as overweight. BMI was calculated using self-reported height and weight from the North Denmark Health Profile 2010.

*Educational status* was included as a categorical variable to indicate the highest completed educational level at baseline and was grouped as follows:

1. Primary (Basic school of <10 years)
2. Secondary (High school education of +3 years or vocational education of +4 years)
3. Higher (Short/medium length higher education of +2 to 4 years or long length higher education of + > =5 years)

Educational data were identified through the Population’s Education Register [39].

*Sleep* was included as a categorical variable. Data were identified through the North Denmark Health Profile 2010 by self-reported hours of sleep in a typical weekday and grouped as less than 7 h, 7 h or more than 7 h of sleep per weekday.

*Household Income* was included and grouped in quartiles. Household income was a measure of the total income in 2009 and was used to the estimate economic status of the respondents at baseline. Household income was identified through the Income Statistics Register [38]. Income was divided by 1.5 when the respondents were registered as living with a partner.

*Previous ulcer* was included as a dichotomous variable based on whether respondents had been either diagnosed with peptic ulcer or received triple treatment before baseline. Diagnoses and triple treatments were identified by the same procedure as the outcome variable. Ulcers were identified as far back as permitted by the registries, i.e., treatments since January 1^st^, 1995 and diagnoses since January 1^st^, 1989.

### Statistics

A *χ* ^2−^test was used to examine baseline characteristics for categorical variables and Student’s *t*-test for continuous variables, with a 0.05 level of significance. Cumulative incidence proportion curves of the first defined peptic ulcer were created; individuals who died during follow-up were censored. Cox proportional hazards regression was used to test the association between stress quintiles at baseline and peptic ulcer within 33 months of follow-up. When calculating the estimates, the stratified sampling design was taken into account using the R-package Svycoxph [45]. A Schoenfeld analysis was conducted to verify the proportional hazard assumption. Triple treatment or diagnosed peptic ulcer were the outcomes of interest and stress quintiles the main exposure. Age, gender, NSAID use, smoking, alcohol consumption, BMI, sleep, educational level, household income and previous ulcer was included in the analysis as covariates. Age was included as a continuous variable after checking the linearity assumption. Tests showed no statistically significant interactions between PSS-10 quintiles and covariates of former ulcer and gender on risk of defined peptic ulcer. Due to the large exclusion of respondents because of missing data on covariates, imputation was performed as a sensitivity analysis. The results based on the imputed data gave similar conclusions and are included in Appendix A and B. A subgroup analysis was performed using only diagnosed peptic ulcers as outcome. Data management was performed using SAS software, version 9.4 (SAS institute Inc. Cary, North Carolina, USA). Statistical analysis was performed using R statistical software package, version 3.2.2 (R Development Core Team).

## Results

**Fig. 1:**
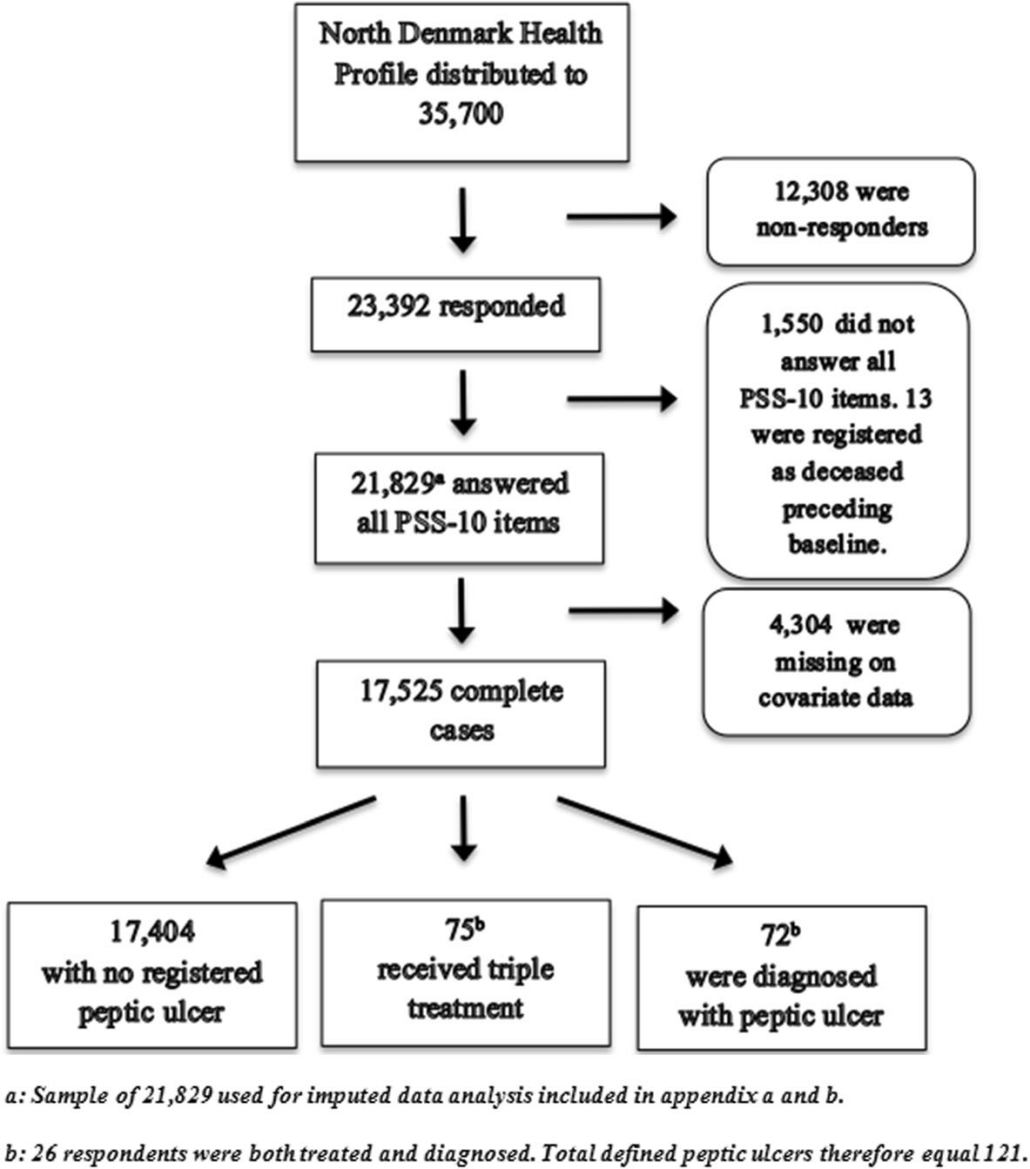
Flow chart from the 35,700 individuals who received the North Denmark Health Profile 2010. The North Denmark Health Profile 2010 was distributed to 35,700 individuals. Individuals who did not respond at all or did not respond to all included covariates were excluded. Final sample size for statistical analysis was 17,525

A total of 35,700 individuals received the health profile questionnaire and there were 12,308 non-responders. There were 1,550 individuals who did not answer all PSS-10 items and were excluded. Preceding baseline, 13 individuals were registered as deceased and were excluded. A total of 4,304 were excluded due to missing data on covariates for the full model adjustment. This left a total sample of 17,525 individuals. During follow-up, 121 defined peptic ulcers were recorded; 75 individuals received triple-treatment whereas 72 were diagnosed in a hospital with a peptic ulcer (26 were both treated and diagnosed, see Fig. 1).

Compared with those with no defined peptic ulcer during follow-up, the individuals with defined peptic ulcers during follow-up were on average 11.1 years older and educated less, and they were more likely to smoke, to use NSAIDs, to sleep less, to earn less income and to have had a defined peptic ulcer before baseline. More than a third of defined peptic ulcers occurred to individuals in the highest stress quintile. There were no signs of significant differences in gender, alcohol consumption or BMI with regards to defined peptic ulcers (Table 2).

**Fig. 2:**
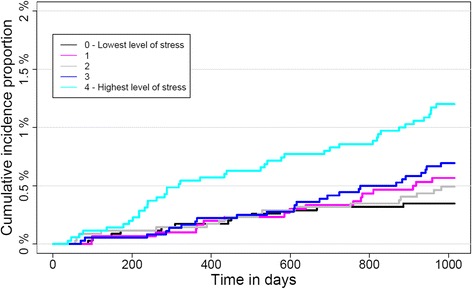
Cumulative incidence proportion of defined peptic ulcers according to self-reported perceived stress level. Cumulative incidence proportion of defined peptic ulcers for a sample of 17,525 Danes participating in the North Denmark Health Profile over time in days for each quintile of the stress-level, as measured by Cohen’s perceived stress scale (PSS-10)

**Fig. 3:**
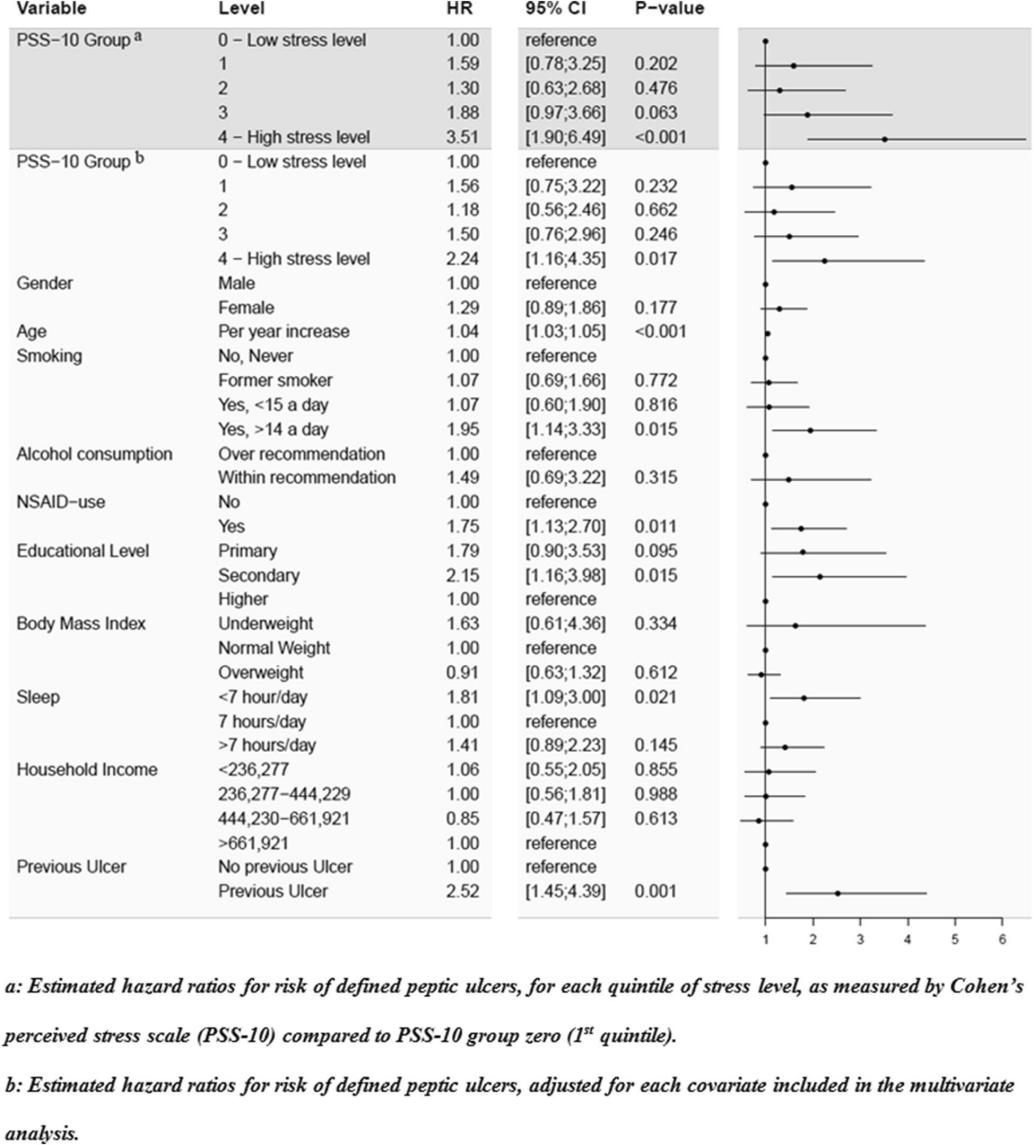
Univariate and multivariate Cox regression model for risk of defined peptic ulcer during follow-up

**Fig. 4:**
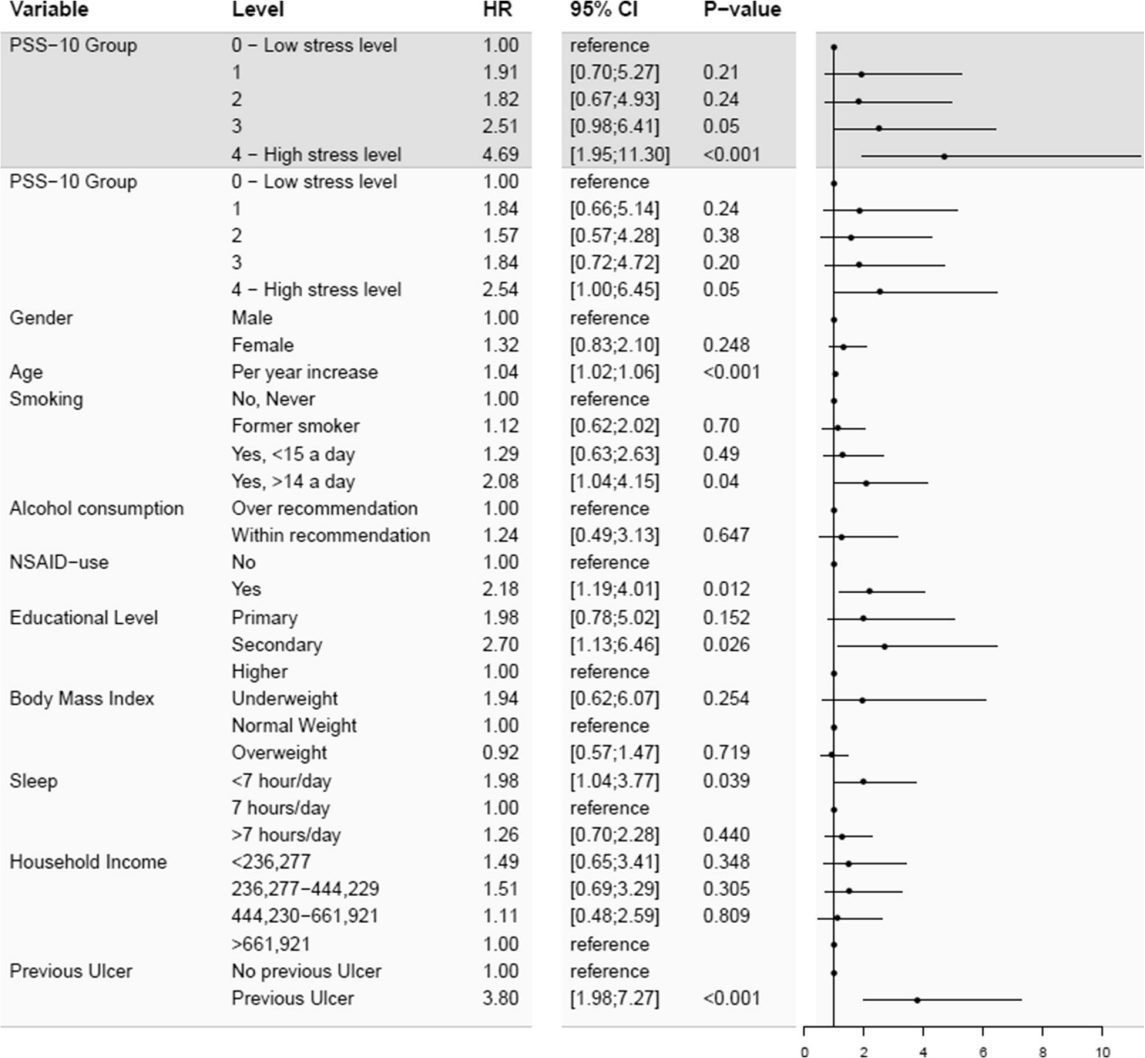
Subgroup analysis of diagnosed peptic ulcer risk during follow-up. Estimated hazard ratios for risk of diagnosed peptic ulcers for each quintile of stress level, as measured by Cohen’s perceived stress scale (PSS-10), compared to PSS-10 group zero (1^st^ quintile). Total sample size was 17,525 individuals

The cumulative incidence proportion of defined peptic ulcers during follow-up is shown in Fig. 2. The curves showed that the highest stress group had the highest incidence proportion of defined peptic ulcers. The highest stress group differed from the lower stress groups continuously throughout follow-up, although it was most evident after approximately 180 days. The risk of defined peptic ulcer was approximately 1.2% in the highest stress group whereas it was approximately 0.4% for the lowest stress group over the 33 months of follow-up. The remaining stress groups were not significantly different from the low stress level during follow up. Figure 3 shows the univariate importance of stress level. Figure 3 also shows the results of a multivariate analysis. The highest stress quintile had a statistically significant higher risk of defined peptic ulcer (HR 3.51 CI 95% 1.90;6.49), compared with the lowest stress quintile at univariate level. The highest stress quintile was at a statistically significant higher risk of defined peptic ulcer (HR 2.24 CI 95% 1.16;4.35), compared with the lowest stress quintile when adjusted for other peptic ulcer risk factors. The remaining stress groups were not significantly different. Older age (HR 1.04; CI 95% 1.03;1.05), more than 14 cigarettes smoked per day (HR 1.95; CI 95% 1.14;3.33), NSAID use (HR 1.75; CI 95% 1.13;2.70), secondary education level (HR 2.15; CI 95% 1.16;3.98), less than 7 h a day of sleep (HR 1.81; CI 95% 1.09;3.00) and previous treatment for or diagnosis with an ulcer (HR 2.52; CI 95% 1.45;4.39) showed significantly increased defined peptic ulcer risk when adjusted for all other covariates. Gender, alcohol consumption, BMI and household income showed no statistically significant differences in peptic ulcer risk after full model adjustment. Imputation of all missing data for all covariates resulted in a sample of 21,829 respondents. Imputation did not affect the significance of the main results; although the hazard ratio for the highest stress quintile in the multivariate model was decreased (HR 2.01; CI 95% 1.18;3.42) (See Appendix A for univariate Cox regression model and Appendix B for multivariate Cox regression model). Subgroup analysis using only diagnosed peptic ulcers as the outcome resulted in increased hazard ratios compared to original analysis. Hazard ratios for highest stress quintile compared to lowest were 4.69 (CI 95% 1.95;11.30) in the univariate model and 2.54 (CI 95% 1.00;6.45) in the multivariate model (Fig. 4).

## Discussion

### Results

This study found that participants with the highest self-perceived stress level had a 2.2-fold higher risk of peptic ulcer treatment in 33 months of follow-up compared to participants with the lowest level of stress. The cumulated incidence of treatment was approximately 1.2% for those with the highest stress levels and 0.4% for those with the lowest levels of stress.

Governmental health agencies in the United States and Denmark claimed that stress was not a cause for peptic ulcer disease [6, 8]. Furthermore, peptic ulcer as a psychosomatic disorder was not consistently supported [29, 31, 32]. Song et al. found no difference in stress level between peptic ulcer patients and controls using the stress severity scale (BEPSI-K) [32], and both Rosenstock et al. and Johnsen et al. found no evidence of peptic ulcers as a psychosomatic disease [22, 31]. However, both studies did not define stress as everyday life stress; Rosenstock et al. used psychological vulnerability and Johnsen et al. used mental depression and coping problems. In contrast, the findings of this study indicated that stress should be considered a determinant of peptic ulcer disease. These findings were supported by several previous studies. Anda et al. found an increased risk of peptic ulcers (OR 1.8) in individuals with self-perceived stress during the month preceding baseline. The study further found evidence of a graded relationship between levels of self-perceived stress and the risk of a peptic ulcer (OR 1.4–2.9) [17]. Our study cannot confirm a graded relationship as only participants in the highest stress quintile were significantly more at risk of developing ulcers compared to participants in the lowest quintile. Anda et al. excluded all respondents with former ulcers [17], whereas this study adjusted for former ulcers as we assumed that the disease was cured after treatment. In current study, stratified analysis based on former ulcers did not suggest a graded relationship and, therefore, it is probably not the reason for the discrepancy. Although Anda et al. also measured perceived stress during the last month, they based the degree of stress on one interview question [17], whereas the current study used 10 items. Wachirawat et al. also found evidence of higher increased odds of a peptic ulcer in patients with high self-perceived stress levels (OR 2.9). However, Wachirawat et al. used a case-referent design, which is particularly prone to information bias [30]. In comparison, this study measured stress before knowledge of the outcome. This strengthens the suggestion that self-perceived stress may cause a peptic ulcer because the ulcer was not what caused the individuals to perceive themselves as stressed. Earlier studies investigating peptic ulcer with stress measured preceding the ulcer also found significant increases in risk. Melinder et al. found that low stress resilience in adolescent males increased the risk of peptic ulcers in adulthood (HR 1.84) compared with high stress resilience [16]. Ruigomez et al. reported increased odds of peptic ulcers (OR 1.58) in a nested case control study among patients who had been diagnosed with stress before their peptic ulcer diagnosis [13], and Levenstein et al. found an increased risk in another Danish sample (OR 2.2) using a stress index preceding 12 years of follow-up.

### Strengths and limitations

As the results were partially based on triple treatment as the outcome measure and respondents were not tested, it was uncertain whether they had an active ulcer or were infected with *H. pylori*. It was recommended for dyspepsia patients with positive *H. pylori* test results to be treated with the same eradication treatment as peptic ulcer patients, and it was estimated that more than half of those patients had an underlying active peptic ulcer [6]. Further, the subgroup analysis, including only diagnosed peptic ulcers yielded similar estimates. Through empirical evidence it had been observed that the effect of *H. pylori* infection on peptic ulcer development was associated to socioeconomic status [29, 30, 46–48], age [2, 30, 47–49] and tobacco smoking [22, 48, 50, 51]. By including these elements in our study analysis we might have diminished the potential confounding effect of the infection on our results; although residual confounding was possibly present. It was, however, unlikely that the perceived stress level should be related to *H. pylori* infection; thus, it was likely evenly distributed among exposed groups rendering it unlikely that confounding by *H. pylori* would be responsible for the results. No research had observed higher infection-rates among stressed individuals which could indicate *H. pylori* infection as a confounder in this study. If *H. pylori* infections should be the reason for the higher risk in the highest stress quintile, then there would have to be some association between stress level and *H. pylori* infection. Rosenstock et al. [52] found that individuals in a Danish sample with *H. pylori* infection had a significantly lower odds ratio for reporting mental stress than those with no infection. If that were the case in the present study, the lower incidence of *H. pylori* infection in the highest stress quintile would result in an underestimated risk of peptic ulcer treatment for the highest stress group.

If individuals who were stressed were more likely to go to their general practitioner when experiencing symptoms than non-stressed, this could be part of the higher treatment risk. Because the follow-up period was 33 months and the increased risk of treatment for the highest stress-level was actually more evident after the first 6 months, confounding by an indication of psychological stress was unlikely in this study.

The validity of the registers used in this study was generally high. The measurement error in the education registers was 0 to 3% [39]. The income statistics register was of high quality and was highly relevant for analysis on economy and health [38]. The use of these registers limited the possibility of information bias as it was not dependent on self-reported data. The registers added power to the analysis as there is no loss to follow-up because there was no need for the respondents to report back themselves. No loss to follow-up also eliminated the risk of selection bias in the follow-up. The questionnaire used to estimate the self-perceived stress level was a validated and often used instrument [40]. The municipality-stratified administration of the North Denmark Health Profile questionnaire increased the generalizability of the results and helped to maintain the large sample size. The non-responders in the North Denmark Health Profile 2010 may be at a higher stress-level than responders if stress was their reason for not responding. This would only result in selection bias if non-responders were also different in peptic ulcer risk, which was considered unlikely in the current study.

## Conclusion

A high perceived stress-level was associated with an increased risk of peptic ulcers. The group with the highest stress level had a 2.2-fold increased risk of having a peptic ulcer compared to the individuals with the lowest stress level. Subgroup analysis of diagnosed peptic ulcer patients found the same risk estimates. The increased risk was not attributable to other risk factors because the effect was not changed substantially by adjustment of known risk factors. These findings disputed the statement in Danish and North American guidelines that everyday life stress as a risk for peptic ulcer is a myth. In contrast, this study indicated that stress is a risk factor for peptic ulcers.

## Appendix A

### Univariate cox regression model of defined peptic ulcer risk during follow-up, imputed dataset

Estimated hazard ratios for risk of defined peptic ulcers for each quintile of stress level, as measured by Cohen’s perceived stress scale (PSS-10), compared to PSS-10 group zero (1^st^ quintile) on an imputed dataset. Total sample size was 21,829 individuals.

## Appendix B

### Multivariate cox regression model of defined peptic ulcer risk during follow-up, imputed dataset

Estimated hazard ratios for risk of defined peptic ulcers adjusted for each covariate included in the multivariate analysis, on an imputed dataset. Data were imputed for all missing values among covariates. Total sample size was 21,829 individuals.

## Abbreviations

ATC: Anatomical therapeutic chemical
BMI: Body mass index
DKK: Danish Kroner, currency
*H. pylori*: *Helicobacter Pylori*
NSAID: Non-steroid anti-inflammatory drugs
Peptic Ulcer: Defined as either receiving triple treatment or being diagnosed with a peptic ulcer during follow-up
PPI: Proton pump inhibitor
PSS-10: Cohen’s perceived stress scale
